# The Rev. Mr. Finch, on Vaccine Inoculation

**Published:** 1800-05

**Authors:** W. Finch

**Affiliations:** Minister of St. Helen's


### *To the Editors of the Medical and Phyfical Journal.*

GENTLEMEN,

ON the 17th of November laft I inoculated a child of eight months old with a lancet, armed the eighth day preceding with vaccine matter, and fent me by the Rev. R. Holt, Rector of Finmere, Oxfordfhire. On the fifth day following, a veficular appearance commenced upon the incifed part; and on the eighth day it became a full puftule. The fymptoms of the fpecific fever appeared the fixth day in the morning, when the child was a little tedious and reftlefs; but not fo much as to give its mother any reafon to fuppofe it was indifpofed from any particular caufe but a flight cold. Upon examining its body, a fmall red fpot was difcovered under its left thigh. It produced no puftule, and began to difappear next day. The puftule on the arm was about a quarter of an inch in diameter; and the efflorefcence around it about an inch and half in diameter. The child had pain in its left axilla.

From the puftule of this child, on the 26th of the fame month, I inoculated fix children; his brother, who was two years and a half old; a girl of feven years and a half old; her brother, of fix years old; a girl three years and a half old; a boy of two years and a quarter old; and an infant four months old. The firft mentioned boy was, at the requeft of his mother, incifed on each arm. On the fourth day after, the veficle appeared on each incifion; and on this day he was affected with a flight indifpofition for about an hour. On the ninth day the puftules were at their full growth. Their fize and the efflorefcence around them were of the fame dimenfions as his brother's. He was affected with pain in both axillæ. The firft girl mentioned, had on the fourth day a flight head-ache, and appeared languid, but was perfectly reftored next morning. Her brother was fo little affected, that, in the language of his parents, they perceived nothing the matter with him; he having, for the moft part, played with his companions out of doors. But upon my inquiring the fifth day, whether, the evening before, he was not more thirfty than ufual? they replied, they thought he was, but could not account for it, unlefs he had got a little cold from being out fo much in the air. This boy had one puftule upon the nape of his neck. The puftules of thefe two children, and the efflorefcence in their arms, and pain in their axillæ, were fimilar to the two above defcribed. The third girl was affected as little as this laft mentioned boy, except having no puftule but on the place of incifion. The boy of two years and a quarter old was much indifpofed for two hours on the fourth day; in every other refpect the effects were of a fimilar nature. It may be remarked of this child, that, from his birth to the time he was inoculated, he was of a feeble habit of body, had not much defire for food, and little inclined to play; but fince, he is grown a very robuft active boy, and eats his meat with a good appetite. His parents are perfuaded it is by virtue of the inoculation that his conftitution is thus invigorated. The infant was indifpofed like the firft child inoculated; but its puftule, when full grown, which was not till the eleventh day, was not above half a quarter of an inch in diameter, and without much efflorefcence.

On the 4th of December, two boys, of fix months old each, were inoculated from a puftule of one of thofe children inoculated culated the 26th of November: One of them had fevar on the third day, and was very reftlefs at even, but next morning appeared as well as ufual. The external fymptoms of the other's indifpofition were tedioufnefs for about three hours in the afternoon of the fourth day, and then he returned to his former fprightlinefs. This child was remarkably lufty. The form of the puftule, the efflorefcence, and pain in the axillæ of thefe two boys were the fame as occurred in the firft child.

The day after I inoculated twenty children of different fexes and ages, from three quarters of a year old to eight years of age. The apparent fymptoms of thofe of three, four, five, fix, and eight years of age, were thirft and chillinefs on the fourth day at even. The reft were indifpofed like thofe of the fame age defcribed above. They were all recovered perfectly the next day. Some of the puftules were grown, on the ninth day, to one-third of an inch in diameter, and the efflorefcence to two inches in diameter. Pain in the axillæ of all, the fame as of the others inoculated before. Of this number, a child of three quarters of a year old had two red fpots, the fifth day, upon the back part of the left fhoulder, but they difappeared the next day without fuppuration. On the left thigh of another of a year old, appeared a red fpot at the fame period, but it went away as the others.

Every ninth or tenth day I have continued inoculating from puftules. The number of my fubjects now amounts to 714. It, perhaps, would be tedious, as well as needlefs, after the above enumeration of cafes, to give a particular detail of the whole, as a judgement may be formed of all from what has been already ftated; this kind of inoculation producing nearly fimilar effects. Suffice it then, as a general account of the external fymptoms that have occurred in this number from the tranfient fever it occafioned, that fome, who were old enough to defcribe their indifpofition, were affected with thirft and chillinefs, generally the fourth day at even. Some infants at the breaft were a little tedious for two or three hours on the fame day, or the day after; and, during this time, made repeated efforts to fuck, and then, upon tafting the milk, as repeatedly declined it. Their mothers obferved to me, that, at this ftage, their mouth and breath were hotter than ufual, and that a little cold water, or cold milk and water, fatisfied them beft. Some patients were drowfy about the time juft fpecified, thought they had got a little cold, and were inclined to go earlier to bed. Some were affected with twitchings in their fleep, and with an increafe of heat on the fkin, in the fourth or fifth night. To this laft cafe there is one exception as to time. An infant of eight months old did not produce vefication till the eighth day; it it then began to be indifpofed, continued fo three days, and was fubject to both the fymptoms juft alluded to for two nights. A few fubjects were fick, and vomited. A girl, of 15 years of age, was affected with dimnefs of fight and difficulty of breathing in the afternoon of the fourth day, and would go to bed fome hours before her ufual time. Her own obfervations were: "Mother, the room is full of fmoke; I cannot breathe; and muft go to bed;" though fhe was affured, at the fame time, by her parent, that the room was perfectly free from vapour. She arofe next morning free from any indifpofition, and fo did they all, except the excepted cafe, and a few whofe indifpofition continued to a part of the next day. I muft alfo obferve, a child of ten months old, fhewing no figns of infection the fifth day, was then inoculated again. On the fifth day after, there being ftill no appearance of inflammation, it was incifed a third time. The day after this laft incifion, a puftule began to form on the left fide of its chin, and was at its full growth the fifth day after; on this day vefication commenced upon the place laft incifed. Another child, of a year and a half old, produced no veficular elation till the fifteenth day after it was inoculated. It was brought to me on the fifth and tenth day; but there ftill appearing a tendency to inflammation, though fcarcely vifible, I deferred incifing it again; and my expectations were at laft fully anfwered. An infant of three months old, not apparently infected by the firft incifion, was, on the ninth day after, inoculated again. A red fpot appeared upon the nape of its neck the day after, grew to a fmall puftule on the fourth day, and was incruftated the feventh day. The puftule on the arm was on the tenth day at its perfection. A few others had one or two red fpots on different parts of their body, but none of them ever fuppurated.

As to the formation of the puftule of the incifed part upon all my fubjects, it varied in its dimenfions. Some puftules were nearly half an inch in diameter; many a quarter of an inch in diameter; and others lefs. The efflorefcent circle defcribed alfo different circumferences: Some fcarcely exceeded the margin of the puftule; fome fpread half an inch in diameter; and fome extended to two, fome to three, fome to four, a few to five inches in diameter, and two nearly met under the arm. And, as an exception to the general affection in the axilla a few fubjects, from ten to fixteen years of age, had pain upon the acromion.

I cannot conclude thefe obfervations without noticing another cafe, peculiar to a young man of about twenty-four years of age. His parents were undecided about his having had the fmall-pox, as he never fhewed any fymptoms of indifpofition, when when the reſt of their children had them naturally. He has always been alarmingly apprehenſive of that diſeaſe; and he was deſirous of putting himſelf to the teſt by inoculation with vaccine matter. On the third morning after I had inoculated him, he was very ſick, and vomitted much; the ſecond day, the inciſed part became elated; on the third was much inflamed: but on the fourth it began to ſubſide, and the fifth day to diſappear. On the tenth day he was again inoculated, but ſtill without any effect. Not ſatisfied, he was, a month after, inciſed again on both arms; but this was attended with no better ſucceſs than before; and he ſeems now perfectly reconciled, that his conſtitution is not ſuſceptible of variolous infection. However, not till after a fourth time of inoculation, could a puſtule be produced upon a child a year and half old, and then on the laſt inciſed part; but it muſt be added, it had no indiſpoſition till the fifth day after the fourth inciſion. And as ſeveral children, who were of a feeble habit of body, did not take the infection upon their firſt inoculation, I preſcribed a better diet than what they had been accuſtomed to. The mothers of thoſe that appeared weakly, and were nurſed at the breaſt, and with whom inoculation was equally unſucceſsful, I adviſed likewiſe to take daily a glaſs of good beer, as preparing their children for the next inſertion. The apparent conſequence was, that every one of them became ſuſceptible of the infection upon a ſecond operation. Its failure, at firſt, ſeemed to be confined to this claſs only; and to two or three others that had been ſeverely burnt. And it may be of importance to remark, that the conſtitution of ſuch ſubjects as appeared feeble before inoculation have ſince been much improved.

For a concluding caſe of my inoculation with vaccine matter:—A mother, who had not had the ſmall-pox, ſubmitted to be inoculated with the vaccine matter, at the ſame time that her infant, which ſhe then ſuckled, was inoculated. He was three months old. As he could be fed with other diet, I cautioned her to decline giving him the breaſt during the time of her indiſpoſition. I had the ſingular pleaſure to experience the happy effects on both.

But, in order to afford a proof of its efficacy to the inhabitants of this place and neighbourhood, on the 25th and 26th of February, I inoculated with variolous matter twenty children, whom I had inoculated with vaccine matter in November and in the beginning of December. The reſult of this experiment is, they have all entirely reſiſted its infection. Inflammation had commenced on the inciſed part the ſecond day, when I ſaw them; and it continued with ſome three, with ſome four, and with others five days, and then began to diſappear. Only two two produced, each, on the place of infertion, a fmall POCK, if fuch it can be denominated, of about a quarter of an inch in diameter: For one was perfectly formed, and difcharged a purulent fluid, on the fourth day. The other generated matter, and alfo threw out purulence the feventh day. The incruftation of the firft is fallen off; that of the other is nearly feparated from its place. This feems to me to be no other a cafe but what nurfes, who have had the fmall-pox, frequently experience, when attendant upon children afflicted with that fore difeafe; and, as in this cafe, they feel no inconvenience from the puftule, fo thefe two patients have not been in the leaft affected. They are brothers. The firft here mentioned is the boy alluded to above, refpecting the improvement of his conftitution; the other, he that was noticed to be remarkably lufty.

Such is the nature, procefs, and effects of inoculation here with vaccine matter; and may ferve as an additional evidence to the teftimony already given of its efficacious properties, if you think it worthy of a place in your very ufeful and inftructive Journal. Upon contemplating its aftonifhing powers, I could not help falling into this reflexion, if the obfervation of the naturalift be true, Wherever a poifonous plant grows, its antidote is found—why may not the fame obfervation hold good in this refpect alfo? If it could be proved, that the human conftitution imbibes that virulent malady, the fmall-pox, from the milk or animal diet, which the lowing herd furnifhes, by parity of reafon then, in the fame quarter its counteracting principle fubfifts. Nature is uniform in all her laws. Every thing fhe generates, has its oppofite. There is no evil without its corrective: And here fhe produces an effectual remedy for that malignancy. It furely cannot be too highly prized. To the difcoverer of it every praife, every gratitude is due. The joy and delight it diffufes throughout every family here is inexpreffible. And it may not be unbecoming to fubjoin, it gives me the livelieft fatisfaction and pleafure to hear the people exclaim—

"Bleffings on the head of him that firft wrought fuch happinefs for us."

I am, GENTLEMEN,

Your moft obedient humble fervant,

W. FINCH,

